# Modulation of Peptide Based Nano-Assemblies with Electric and Magnetic Fields

**DOI:** 10.1038/s41598-017-02609-z

**Published:** 2017-06-02

**Authors:** Gaurav Pandey, Jahnu Saikia, Sajitha Sasidharan, Deep C. Joshi, Subhash Thota, Harshal B. Nemade, Nitin Chaudhary, Vibin Ramakrishnan

**Affiliations:** 1 Department of Biosciences and Bioengineering, Indian Institute of Technology Guwahati, Guwahati, 781039 India; 2 Department of Physics, Indian Institute of Technology Guwahati, Guwahati, 781039 India; 3 Department of Electronics and Electrical Engineering, Indian Institute of Technology Guwahati, Guwahati, 781039 India

## Abstract

Peptide based nano-assemblies with their self-organizing ability has shown lot of promise due to their high degree of thermal and chemical stability, for biomaterial fabrication. Developing an effective way to control the organization of these structures is important for fabricating application-oriented materials at the molecular level. The present study reports the impact of electric and magnetic field-mediated perturbation of the self-assembly phenomenon, upon the chemical and structural properties of diphenylalanine assembly. Our studies show that, electric field effectively arrests aggregation and self-assembly formation, while the molecule is allowed to anneal in the presence of applied electric fields of varying magnitudes, both AC and DC. The electric field exposure also modulated the morphology of the self-assembled structures without affecting the overall chemical constitution of the material. Our results on the modulatory effect of the electric field are in good agreement with theoretical studies based on molecular dynamics reported earlier on amyloid forming molecular systems. Furthermore, we demonstrate that the self-assemblies formed post electric-field exposure, showed difference in their crystal habit. Modulation of nano-level architecture of peptide based model systems with external stimulus, points to a potentially rewarding strategy to re-work proven nano-materials to expand their application spectrum.

## Introduction

Ability to design, control morphology, and tune up physical and chemical properties at nanoscale characterize the heart of nanotechnology. Generation of nano-level architecture should ideally have a design phase at molecular scale. Morphology of such systems can be better controlled, if they assemble further-on to micro dimensions. Nanotechnology research mostly focuses on the latter half by way of their imminent utility while fabricating materials at larger dimensions. Application of physical agents for tailoring the nanostructure morphology can be very useful for nanostructure fabrication.

In last decade, there has been an increased focus on organic and bio-organic nano-assemblies. Peptide nanotubes, their physical properties, and assembly morphologies are extensively studied due to their excellent biocompatibility as well as functional and structural diversity. Many ordered supramolecular structures have been constructed using peptides as the building blocks. The most extensively utilized peptide-based building block is diphenylalanine (Phe-Phe or FF), which is the shortest bio-molecule known to self-assemble into ordered nanostructures. FF incidentally is also the core recognition motif of the β-amyloid polypeptide, a peptide associated with Alzheimer’s disease^1^. It can self-assemble into a variety of structures like microtubes, nanotubes^2^, microcrystals, nanofibers^3^, nanorods^4, 5^ and nanowires^6^.

The potential of these supramolecular structures have been utilized in diverse fields including nanofabrication, drug delivery vehicles^7^, bio-sensing^8^, energy storage devices, and hydrogels for tissue engineering^9, 10^. The crystal structure of FF exhibits a non-centrosymmetric hexagonal space group (P6_1_)^1, 11^, which allows it to possess properties like piezoelectricity^12, 13^, optical activity^14, 15^ and ferroelectricity^16^. Due to its low dimensional highly ordered structure it also exhibits quantum confinement^17^, forming quantum dots^18^. Self-assembled structures formed by analogues of FF such as Ac-Phe-Phe-NH_2_, NH_2-_Phe-Phe- NH_2_, NH_2_-(*p*-nitro-Phe)-(*p*-nitro-Phe)-COOH, NH_2_(4-phenyl-Phe)-(4-phenyl-Phe)-COOH, PEGylated tetra-phenylalanine (L6-F4), β-AspFF etc. have also resulted in tubular, fibrillar and squared plate structures respectively^10, 19, 20^.

One of the key challenges in the field of supramolecular chemistry has been controlling the self-assembly of molecules into ordered functional units. Previously, a number of strategies including pH mediated control^21, 22^, solvent mediated control^23^, covalent modifications^24^, vapour deposition^25, 26^, temperature^27^, surface^28, 29^, relative humidity^30^, symmetry^31^ and magnetite coating on the surface of nanotubes^32^ have been employed to regulate the architecture of diphenylalanine self-assemblies. But studies with fairly high magnetic fields have not yielded the level of response as expected, though a response was forced, at a magnetic field range of 12 T or more^33^. Recent theoretical investigations, predict measurable response to applied electric field but practical demonstration has not been reported so far, with the best of our knowledge. The present study aims to investigate the impact of electric and magnetic field mediated perturbation, upon the chemical and physical properties of peptide nano assemblies, with diphenylalanine as the model molecular system. Electric field studies on biomolecules (like DNA and proteins) have shown permanent or induced dipole formation along the direction of applied field, indicating that electric field interaction could modulate their dipole moments^34, 35^. Molecular dynamics studies in the presence of external electric fields on Aβ amyloid peptides and insulin have conclusively predicted the effect of an external field in modulating structure formation^12, 36^. Martin Garcia and Ojeda May have predicted that an external constant electric field can even modify the secondary structure of a protein, by inducing a transition from β-sheet to α-helical like conformation^37^. Andrij Baumketner in a recent study, explored the feasibility of using external electric field to disaggregate amyloid fibrils, by inducing folding into an α-helical state reducing their β sheet conformation^38^. This is especially important because FF is the core recognition motif of β-amyloid segment. Here in this study, we attempt to confirm the effect of AC (Alternating Current) and DC (Direct Current) electric field on diphenylalanine self-assembly using experimental approach.

Protein crystals have been reported to show alignment in the presence of strong magnetic field. This alignment was believed to be the result of diamagnetic anisotropy of the peptide bonds and aromatic amino acids in protein molecules^39^. These aromatic side chains play an important role in directing the amyloid fibril self-assembly through π-π stacking interactions^40^. Magnetic field has been previously employed to align β-amyloid fibrils for X-ray fiber diffraction studies^41–44^. So far various groups have reported studies involving magnetic field exposure of self-assembled diphenylalanine assemblies post formation^32, 33^. We, however use electric or magnetic field, concurrently while formation of nano-assemblies. This is achieved by starting the experiment at a temperature (95 °C) that do not support assembly. The system is then cooled to facilitate assembly under electric or magnetic field of chosen strength at desired optimum concentrations for assembly. Our results indicate that field induced perturbation approach can be a promising tool for controlling nano-assembly, thus generating novel architectures.

## Results and Discussion

### Electric and magnetic field effects on nano-assembly

**Figure 1:**
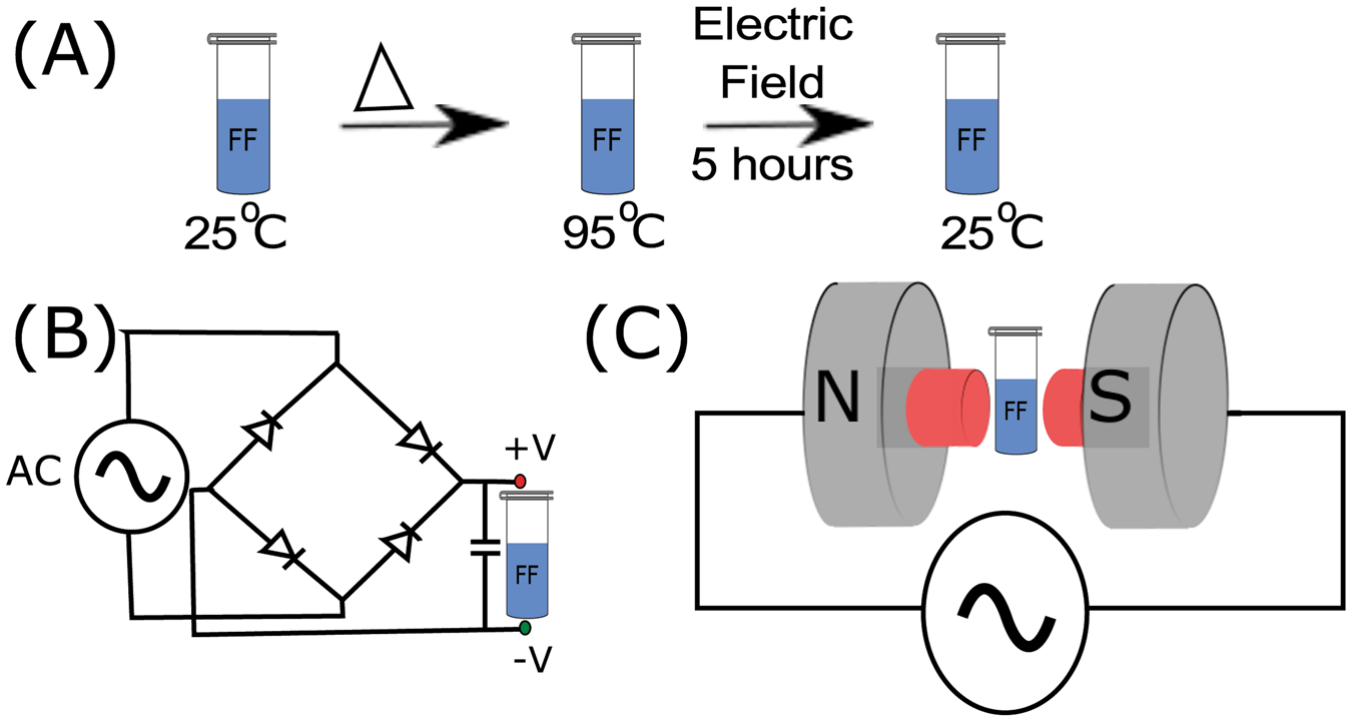
Schematic representation of experimental setup. (**A**) Dissolved peptide was allowed to cool down from 95 °C to room temperature while incubating in the presence of electric field. (**B**) Electric field set up and (**C**) magnetic field setup.

The experimental system containing the peptide sample was allowed to cool in the presence of electric (150 Vcm^−1^/50 Hz, 300 Vcm^−1^/50 Hz AC electric field, and 150 Vcm^−1^, 300 Vcm^−1^ DC) fields, magnetic field and ambient conditions continuously for 5 hours till the system cools and gets equilibrated at room temperature (25 °C) completely (Fig. 1). Interestingly, there was no visible self-assembly as long as the sample was under electric field exposure. The control sample with no field, and the sample cooled under magnetic field (0.6 T), showed visible nano-assemblies, which was further confirmed by light scattering and FE-SEM experiment. These observations suggest that both AC as well DC electric fields could inhibit an undirected aggregation process and a directed self-assembly process.

**Figure 2:**
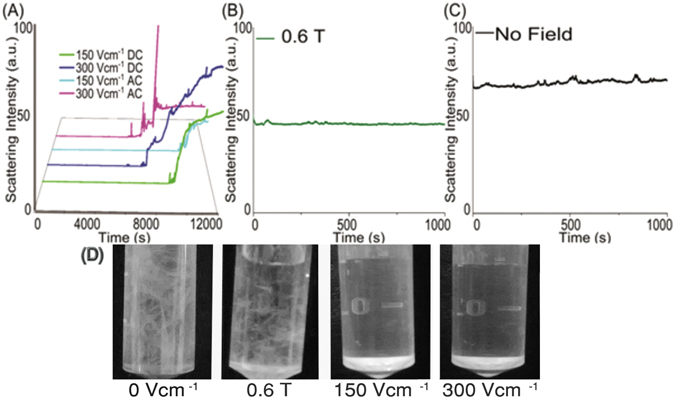
Effect of the electric and magnetic field on FF nano-assembly. The graphs represent static right angle scatter plots post 5 hours’ incubation at (**A**) electric field, (**B**) magnetic field, (**C**) no field mentioned conditions in arbitrary units (a.u.) and (**D**) post annealing under various field conditions: Images were taken after cooling the FF solution for 5 hours. Electric field (150 Vcm^−1^ and 300 Vcm^−1^ clearly has a prohibitive effect on aggregation and nano-assembly of FF; whereas magnetic fields at 0.6 T and 0 V (no field) show a dense growth of visible self-assembled FF aggregates.

Previously, it has been shown that FF tubes align in the presence of a very high magnetic field (12 T)^33^, perhaps magnetic fields as low as 0.6 T was not robust enough to perturb the forces involved in the self-assembly process. Static light scattering was recorded at right angles for each of the sample to quantify the aggregation in FF annealed under an electric and magnetic field in comparison to the control sample, immediately after an incubation of 5 hours (Fig. 2). Time axis shown in Fig. 2 represents the time period for which static light scattering measurements were made.

The right angled static light scatter of the samples exposed to AC and DC electric field showed an increase in scattering intensity with time, indicating spontaneous initiation of peptide self-assembly (Fig. 2A). In contrast, the control and magnetic field exposed samples showed very high scattering intensity at zero time signifying the presence of pre-formed self-assemblies (or aggregates) at appreciable dimensions (Fig. 2B,C). For comparing this phenomenon, peptide solution was cooled from 95 to 25 °C and its right angled static light scatter was recorded as a control experiment. This experiment also showed a pattern similar to electric field exposed samples, and the right-angled static light scattering showed an increase with time, as a result of the spontaneous initiation of peptide self-assembly upon cooling (Supplementary Fig. S1).

### Effect of the electric field in modulating nanotube morphologies

**Figure 3:**
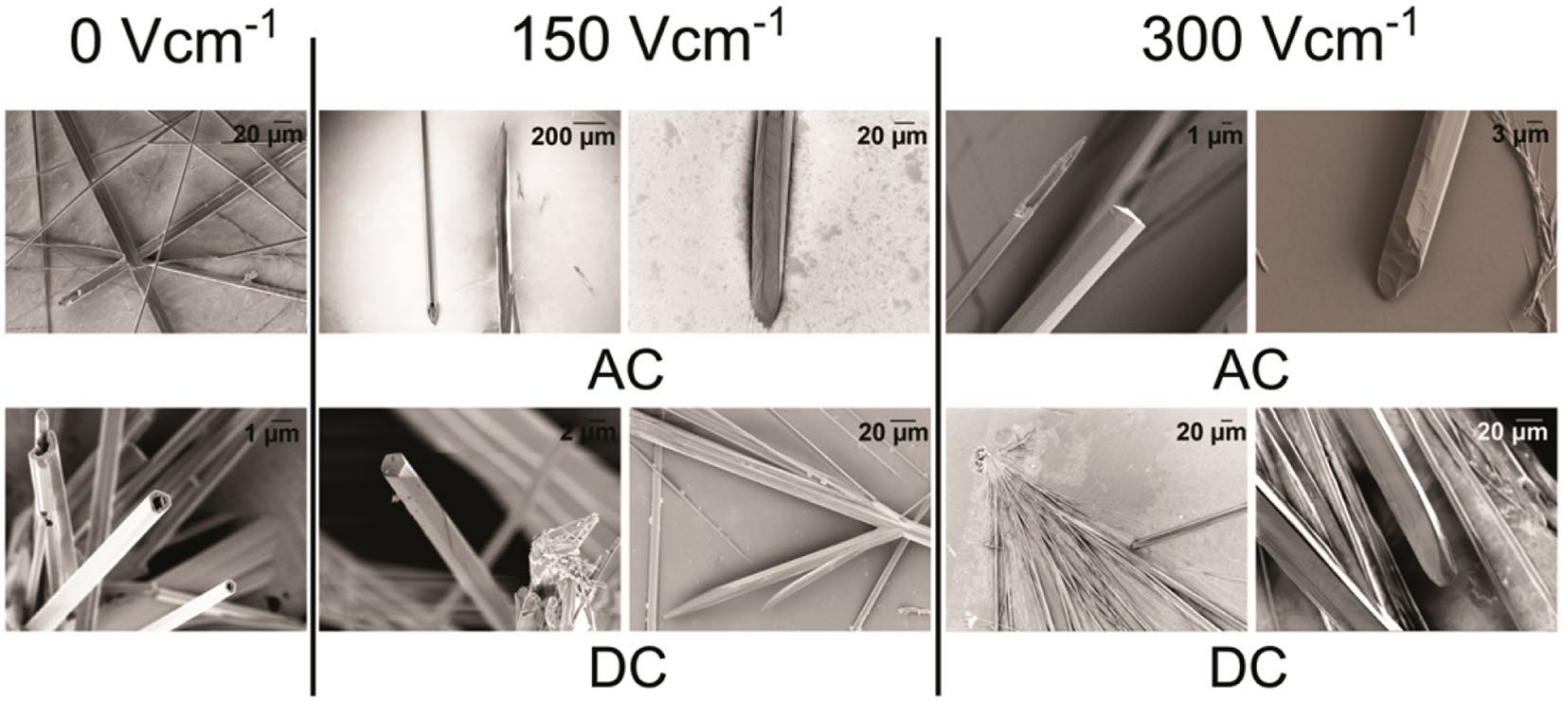
Scanning electron micrographs of FF self-assemblies formed post annealing at room temperature conditions: The nanorods and nanotubes formed after five experimental conditions reported (0 V cm^−1^, 150 V cm^−1^ DC and AC electric field; 300 Vcm^−1^ AC and DC electric field) are shown. Field induced assemblies have greater tendencies of alignment and a morphological shift to rod like structures (compared to tubes) in the overall population of nano-micro dimensional structures.

The samples were analyzed using FE-SEM to gain an insight into the effect of electric field on the peptide self-assemblies, (Fig. 3 and Supplementary Fig. S2). Morphologically the self-assemblies formed post AC, and DC electric field exposures were more aligned with the presence of branched structures. They also showed a morphological shift to rod-like structures, though tubes were also observed. The self-assemblies formed in the control sample (0 Vcm^−1^) formed an exclusive tube-like morphology and were randomly oriented in comparison to the field samples. The results indicate the possibility that electric field exposure modulates the formation of the peptide self-assemblies, though the magnitude of this influence may vary with sample molecular systems.

To investigate the effect of electric field exposure upon the physical properties of peptide self-assemblies, melting points of each sample were measured. The samples showed the difference in their melting points, further confirming a modulation in the assembly architecture of FF nanostructures (Table 1).

**Table 1:** Melting points for diphenylalanine nano-assembled tubes/rods after a 5 hours exposure to specific DC electric fields in comparison to control.

Sample	Melting Point (°C)
0 Vcm^−1^	288–291
150 Vcm^−1^	284–285
300 Vcm^−1^	278–280
FF powder (sigma)	291–292

### Effect of an external stimulus on the chemical constitution

**Figure 4:**
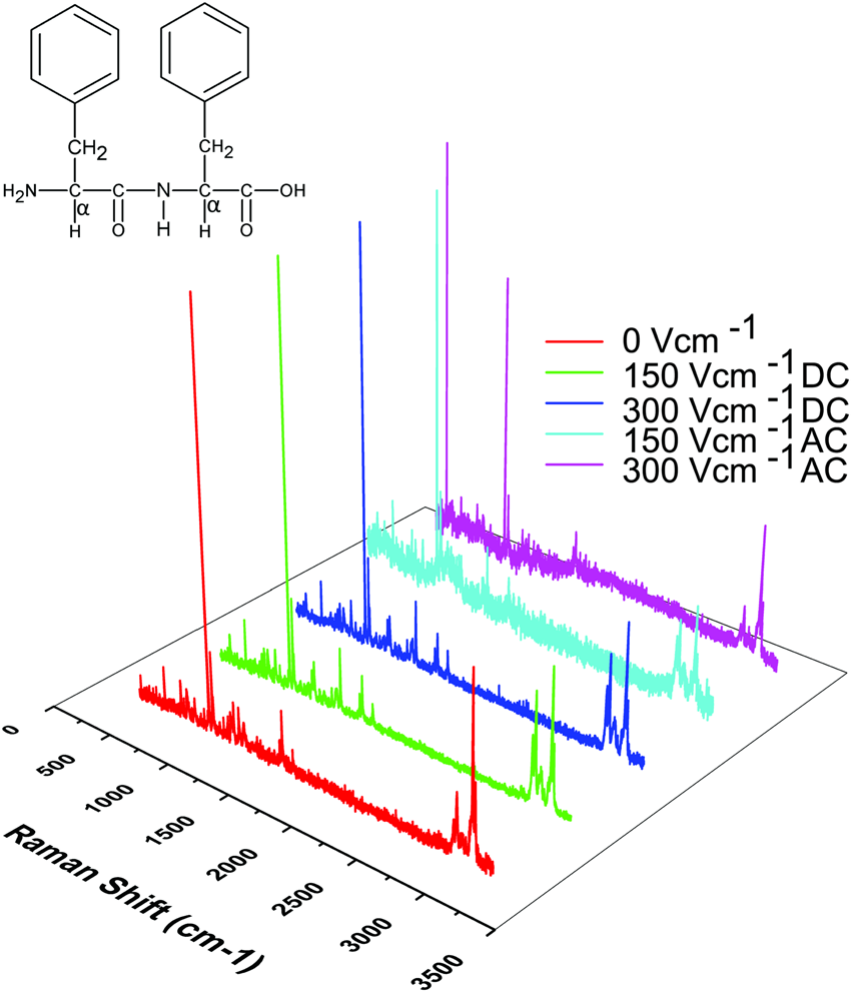
Chemical constitution from Raman spectra. Raman spectra were recorded at five different experimental conditions (0 Vcm^−1^, 150 V cm^−1^ DC and AC electric field; 300 Vcm^−1^ AC and DC electric field). Spectral analysis suggests that chemical constitution of all five samples are identical, indicating that field effect is modulating only the assembly formation at nano-level.

High-resolution micro-Raman spectroscopy is a non-invasive technique that can be employed to examine the functional group composition of the material. This technique was performed on dried diphenylalanine self-assemblies, from each of the experimental conditions to probe the impact of field exposure on the chemical composition of the peptide. The Raman spectra of the self-assemblies formed in control and field exposed samples are shown in Fig. 4.

The Raman bands correspond to aromatic rings of FF are at 1004 cm^−1^, 1034 cm^−1^, 1590 cm^−1^, 1429 cm^−1^ and 1608 cm^−1^ ^45, 46^ Peak at 1688 cm^−1^ band can be attributed to C=O stretching while small peaks detected between 1154 to 1300 cm^−1^ can be assigned to the acyclic C-C stretching^45, 46^. Supplementary Table S1 lists the Raman shifts of chemical functional groups of diphenylalanine. Similar characteristic bands were observed in each of the samples, and there was no remarkable difference molecule is not affected by the field induced perturbation. Therefore, the difference in assembly, morphology and melting points may be attributed to the modulatory effect of electric field on assembly formation.

To confirm the phase purity and examine any possible difference in crystal structure on the self-assemblies formed with and without electric field exposure, powder X-ray diffraction (PXRD) measurements were performed with Cu-Kα radiation (λ = 1.54 Å). Le-Bail profile fitting has been performed by assuming hexagonal crystal structure symmetry with P61 space group^47^.

**Figure 5:**
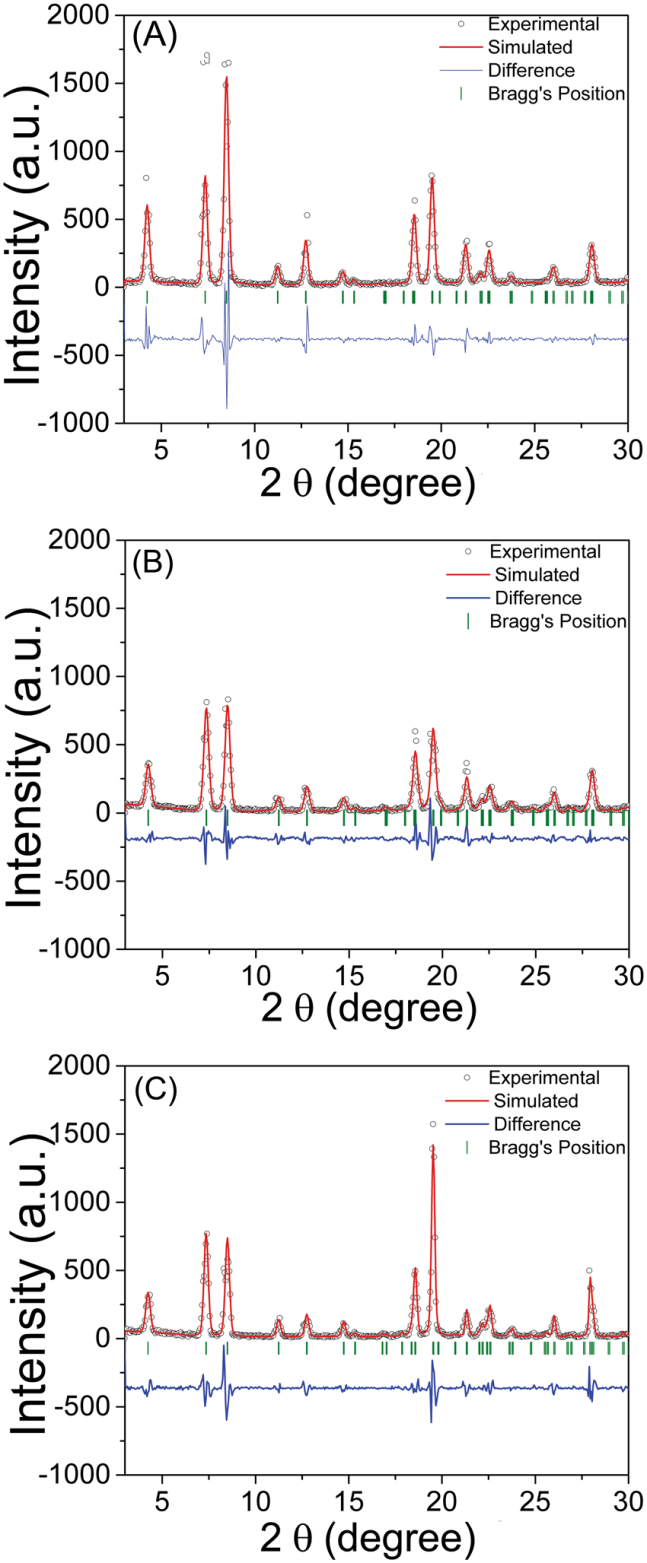
X-ray powder diffractogram of FF self-assemblies at (**A**) ambient conditions, post DC electric field incubations at (**B**) 150 Vcm^−1^ and (**C**) 300 Vcm^−1^. Hollow dots: measured powder diffraction pattern after background subtraction; solid red curve: simulated profile; green vertical tick marks: Bragg’s positions; and the solid blue curve: residual between the experimental and simulated profiles.

A pseudo-Voigt (P-V) function was used to fit the profile by using Full Prof Suite^48^. P-V is an approximation of Voigt function, which is a linear combination of the Gaussian profile and Lorentzian profile^49^, ^50^. This function allows to accommodate asymmetries in the X-ray diffraction peaks. The scattered points in Fig. 5 shows the experimentally observed diffraction data and the red colour solid line represent the Le-Bail profile fitting of diphenylalanine polycrystalline samples exposed to electric field. These X-ray diffraction patterns are in-agreement with hexagonal structure proposed by Gorbitz and the resultant crystallographic data is consistent with the published FF crystal structure (CCDC 163340)^1^. No spurious peaks were observed in the X-ray diffraction pattern which confirms the formation of pure diphenylalanine.

**Figure 6:**
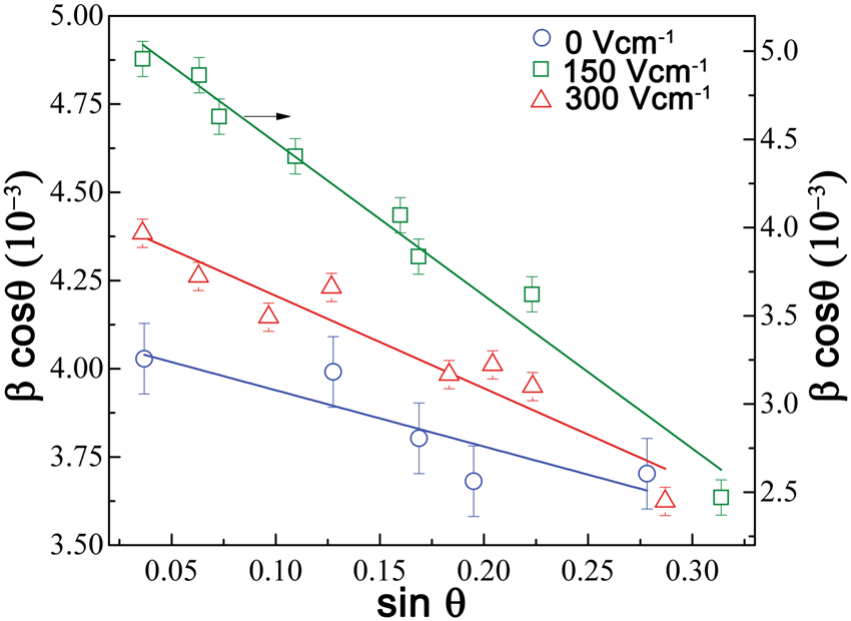
The Williamson-Hall plots (β cosθ versus sinθ) of FF self-assemblies formed at ambient conditions. The hollow blue, olive and red color symbols represent the post-DC electric field incubation of 0 Vcm^−1^, 150 Vcm^−1^and 300 Vcm^−1^, respectively.

The Le-Bail profile fitting of X-ray diffraction patterns also showed that all three samples differ with respect to their lattice parameters. Average crystallite size ‘P’ and micro strain ‘η’ play a significant role in the peak broadening^51^. In order to estimate the individual contribution of ‘P’ and ‘η’ on peak broadening, the Williamson Hall (W-H) analysis was employed (Fig. 6).

The W-H analysis is governed by the following relation with which we can estimate the contribution of crystallite size ‘P’ and lattice strain ‘η’ on X-ray peak broadening:

$$\beta \,\cos \,\theta =\frac{{\rm{K}}\lambda }{{\rm{P}}}+\eta \,\sin \,\theta $$

where, β is the full-width half maximum of diffraction peak intensity, K (~0.89) is the shape factor, and λ is the wavelength of Cu-Kα X-ray radiation.

Crystallite size (P~ 322–342 Å) of the particles gives a rough estimation of the domain size which diffract coherently. It is mostly not the same as particle size due to the formation of polycrystalline aggregates^52^.

Lattice strain on the other hand quantifies the distribution of lattice constants originating from imperfections in the crystals, such as dislocations inside the lattice. Figure 6 shows the W-H plot (β cosθ versus sinθ) for FF under different electric fields. From the slope and intercept of these plots, the magnitude of ‘P’ and ‘η’ was estimated. The self-assemblies formed post-electric field exposure differ significantly on average crystallite size as well as lattice strain. All the crystallographic parameters for the FF obtained from the profile fitting are listed in Table 2.

**Table 2:** Lattice parameters derived from Le-Bail profile fitting and W-H analysis of X-ray diffraction patterns of diphenylalanine self-assembled structures.

	0 Vcm^−1^	150 Vcm^−1^	300 Vcm^−1^
a	24.069124	24.069912	24.052418
b	24.069124	24.069912	24.052418
c	5.407554	5.402272	5.454072
α	90°	90°	90°
β	90°	90°	90°
γ	120°	120°	120^0^
P	322 Å	403 Å	343 Å
η	2.2 × 10^−3^	6.2 × 10^−3^	4 × 10^−3^

## Conclusion

We demonstrated that electric field could arrest the hierarchical self-assembly of diphenylalanine, and self-assembly was initiated only after (at least 10 minutes) the withdrawal of external electric field whereas magnetic field at the experimental (0.6 T) range has no significant effect on assembly formation. FF is the core recognition motif of amyloid β polypeptide. Electric field effect is in agreement with the earlier observation made by Andrij Baumketner, that external electric field can disaggregate amyloid fibrils by inducing folding. Self-assembly of diphenylalanine peptide after annealing in the presence of electric field showed the distinct morphological difference. Hill and co-workers earlier studied the little effect of magnetic field, and the results are in full agreement with our observation. The electric field exposure also modulated the stability of the self-assembled structures without affecting the overall chemical constitution of the material. Furthermore, we demonstrated that the self-assemblies formed post electric-field exposure showed the difference in their crystal habit. W-H analysis of the X-ray diffraction patterns revealed the differences in the peptide self-assemblies formed post electric field exposure of varying magnitudes, with respect to their lattice parameters as well as crystallite size. The microstrain associated with self-assembled structures also varied with changes in the magnitude of the electric field. We infer that our strategy in employing electric field mediated perturbation of electrostatic interactions directing the peptide self-assembly can be used to generate nanostructures with distinct morphologies from the same material. Further, in line with the theoretical investigations discussed in the earlier sections, this experiment suggests that electric field can modulate the physical properties of nanostructures and therefore has the potential to be deployed as a fabrication tool for a myriad of application-oriented materials without any chemical additives.

## Materials and Methods

The FF peptide was purchased from Sigma-Aldrich. Deionized water was used for all the experiments.

### Preparation of Peptide Solution

Fresh solution of diphenylalanine peptide was prepared by dissolving the lyophilized form of the peptide in deionized water (at 95 °C) to a concentration of 2 mg/ml^27^. The vial containing the sample was kept in a water bath (Grant sub aqua 26 plus) set at 95 °C, allowing peptide to dissolve completely. Fresh stock was prepared for each experiment to avoid any pre-aggregation. Peptide nano/micro tubes were formed while cooling to room temperature (25 °C).

### Pre-aggregation test

To ensure the absence of any pre-aggregation in peptide stock solution a right angle scatter was recorded using a spectrofluorometer (Jasco FP 8500) at 450 nm. The slit width was set at 2.5 nm^53^.

### Electric Field Experiments

The experiment was performed with FF at a concentration of 2 mg/ml at room temperature (25 °C) for a duration of 5 hours, at three different DC and AC electric fields: 0 Vcm^−1^ (control), 150 Vcm^−1^ and 300 Vcm^−1^ (Fig. 1A). Two parallel aluminum electrodes separated by a distance of 1 cm were anchored to a plate to produce a horizontal DC electric field (Fig. 1B). These plates were connected to a full-wave bridge rectifier, and a single-phase variable autotransformer was used to regulate the voltage and to produce a horizontal AC electric field a similar setup was built without a full-wave bridge rectifier. A 2 ml of FF peptide solution (at 95 °C) was taken in a microcentrifuge tube and allowed to cool in the presence of electric field for a duration of 5 hours. No voltage was applied between the electrodes in the case of control experiment. All the experiments were done in parallel, to ensure that sample allowed to cool under electric field (AC as well as DC), magnetic field and control (no field) conditions cooled at same rate.

### Magnetic Field Experiment

Similar to electric field experiment, a magnetic field experiment was also performed with 2 mg/ml FF solution at room temperature for a duration of 5 hours. Peptide solution (95 °C) was taken in a microcentrifuge tube and allowed to cool in the presence of magnetic field measuring 0.6 T (Fig. 1C).

### Static Right Angle Light Scattering Assay

Static light scattering was recorded for each of the sample to quantify the aggregation in FF annealed under an electric and magnetic field in comparison to the control sample immediately after an incubation of 5 hours, using a spectrofluorometer (Jasco FP 8500) at 450 nm. The slit width was set at 2.5 nm^53^. Post electric annealing, field-exposed peptide solution was taken in a fluorescence cuvette (Helma, Sigma-Aldrich) of 1 cm path length, and its static light scattering was recorded immediately for 12000 seconds. For control as well as magnetic field sample which already comprised dense FF aggregates, static light scattering was recorded only for 1000 seconds. The term “post incubation/annealing” refers to the time period after 5 hours, when both electric as well as magnetic field were switched off.

### Field Emission Scanning Electron Microscopy (FE-SEM)

FE-SEM analyses were performed using a Zeiss Sigma FE-SEM at 2–3 kV. Field exposed peptide samples were loaded on a glass slide for analyses and air dried. Samples were coated with gold for enhancing conductivity.

### Melting Point Estimation

Melting points for the diphenylalanine self-assemblies formed under different experimental conditions were recorded using Optics Technology Digital Melting Point Apparatus. The solid samples (FF self-assemblies) were packed in thin glass capillaries and loaded in the apparatus. For each sample, the temperature at which the first drop of liquid appears was recorded as the melting point of that sample.

### Raman Spectroscopy

Raman measurements of dried FF self- assemblies were performed using a high-resolution micro-Raman spectrometer (Jobin Horiba, LabRam HR800); 514 nm laser was used for all the experiments.

### Powder XRD

X-Ray diffraction (XRD) patterns were obtained from a Rigaku X-ray diffractometer (Model: TRAX III) powder diffractometer operating at 50 kV and 100 mA using Ni-filtered Cu-Kα radiation (λ = 1.54 Å). The diffractograms were recorded in the 2θ range of 0–30°.

## Electronic supplementary material

Supporting information

## References

[1] Görbitz, C. H. The structure of nanotubes formed by diphenylalanine, the core recognition motif of Alzheimer’s β-amyloid polypeptide. *Chem. Commun*. 2332–2334 (2006). 10.1039/b603080g 16733570

[2] Bong DT, Clark TD, Granja JR, Ghadiri MR (2001). Self‐assembling organic nanotubes. Angew. Chem., Int. Ed. Engl..

[3] Nalluri SKM (2014). Conducting nanofibers and organogels derived from the self-assembly of tetrathiafulvalene-appended dipeptides. Langmuir.

[4] Li Q, Jia Y, Dai L, Yang Y, Li J (2015). Controlled Rod Nanostructured Assembly of Diphenylalanine and Their Optical Waveguide Properties. ACS Nano.

[5] Ziganshin MA (2016). Thermally induced diphenylalanine cyclization in solid phase. J. Therm. Anal. Calorim..

[6] Reches M, Gazit E (2003). Casting metal nanowires within discrete self-assembled peptide nanotubes. Science.

[7] Silva RF, Araujo DR, Silva ER, Ando RA, Alves WA (2013). L-diphenylalanine microtubes as a potential drug-delivery system: characterization, release kinetics, and cytotoxicity. Langmuir.

[8] Sousa CP, Coutinho-Neto MD, Liberato MS, Kubota LT, Alves WA (2014). Self-assembly of peptide nanostructures onto an electrode surface for nonenzymatic oxygen sensing. The J. Phys. Chem. C.

[9] Smith AM (2008). Fmoc‐Diphenylalanine Self Assembles to a Hydrogel via a Novel Architecture Based on π–π Interlocked β‐Sheets. Adv. Mater..

[10] Xue, P. *et al*. Cation Tuning toward the Inference of the Gelation Behavior of Supramolecular Gels. *Sci. Rep*. **6** (2016). 10.1038/srep25390 PMC4853806 27138527

[11] Görbitz CH (2001). Nanotube formation by hydrophobic dipeptides. Chem. - Eur. J..

[12] Kelly CM (2015). Conformational dynamics and aggregation behavior of piezoelectric diphenylalanine peptides in an external electric field. Biophys. Chem..

[13] Nguyen V, Zhu R, Jenkins K, Yang R (2016). Self-assembly of diphenylalanine peptide with controlled polarization for power generation. Nat. Commun..

[14] Yan X, Li J, Möhwald H (2011). Self‐Assembly of Hexagonal Peptide Microtubes and Their Optical Waveguiding. Adv. Mater..

[15] Ryu J, Lim SY, Park CB (2009). Photoluminescent peptide nanotubes. Adv. Mater..

[16] Gan Z, Wu X, Zhu X, Shen J (2013). Light‐Induced Ferroelectricity in Bioinspired Self‐Assembled Diphenylalanine Nanotubes/Microtubes. Angewandte Chemie International Edition Angew. Chem., Int. Ed..

[17] Amdursky N, Gazit E, Rosenman G (2010). Quantum Confinement in Self‐Assembled Bioinspired Peptide Hydrogels. Adv. Mater..

[18] Amdursky N, Molotskii M, Gazit E, Rosenman G (2009). Self-assembled bioinspired quantum dots: optical properties. Appl. Phys. Lett..

[19] Reches M, Gazit E (2006). Designed aromatic homo-dipeptides: formation of ordered nanostructures and potential nanotechnological applications. Phys. Biol..

[20] Diaferia, C. *et al*. Self-assembly of PEGylated tetra-phenylalanine derivatives: structural insights from solution and solid state studies. *Sci. Rep*. **6** (2016). 10.1038/srep26638 PMC4879547 27220817

[21] Tang C, Smith AM, Collins RF, Ulijn RV, Saiani A (2009). Fmoc-diphenylalanine self-assembly mechanism induces apparent p K a shifts. Langmuir.

[22] Martins TD (2011). Influence of pH and pyrenyl on the structural and morphological control of peptide nanotubes. The J. Phys. Chem. C..

[23] Mason TO (2014). Expanding the solvent chemical space for self-assembly of dipeptide nanostructures. ACS nano.

[24] Gour N, Barman AK, Verma S (2012). Controlling morphology of peptide‐based soft structures by covalent modifications. J. Pept. Sci..

[25] Chen J, Qin S, Wu X, Chu KP (2015). Morphology and Pattern Control of Diphenylalanine Self-Assembly via Evaporative Dewetting. ACS nano.

[26] Vasudev MC (2014). Vertically aligned peptide nanostructures using plasma-enhanced chemical vapor deposition. Biomacromolecules.

[27] Huang R, Wang Y, Qi W, Su R, He Z (2014). Temperature-induced reversible self-assembly of diphenylalanine peptide and the structural transition from organogel to crystalline nanowires. Nanoscale Res. Lett..

[28] Huang R, Qi W, Su R, Zhao J, He Z (2011). Solvent and surface controlled self-assembly of diphenylalanine peptide: from microtubes to nanofibers. Soft Matter.

[29] Demirel G, Buyukserin F (2011). Surface-induced self-assembly of dipeptides onto nanotextured surfaces. Langmuir.

[30] Wang M, Du L, Wu X, Xiong S, Chu PK (2011). Charged diphenylalanine nanotubes and controlled hierarchical self-assembly. ACS Nano.

[31] Sasidharan S, Hazam PK, Ramakrishnan V (2017). Symmetry-Directed Self-Organization in Peptide Nanoassemblies through Aromatic π–π Interactions. The J. Phys. Chem. B.

[32] Reches M, Gazit E (2006). Controlled patterning of aligned self-assembled peptide nanotubes. Nat. Nanotechnol..

[33] A Hill R (2007). Alignment of aromatic peptide tubes in strong magnetic fields. Adv. Mater..

[34] Diekmann S, Hillen W, Jung M, Wells RD, Pörschke D (1982). Electric properties and structure of DNA restriction fragments from measurements of the electric dichroism. Biophys. Chem..

[35] Pörschke D (1987). Electric, optical and hydrodynamic parameters of lac repressor from measurements of the electric dichroism High permanent dipole moment associated with. Biophys. Chem..

[36] Lugli F, Toschi F, Biscarini F, Zerbetto F (2010). Electric Field Effects on Short Fibrils of Aβ Amyloid Peptides. J. Chem. Theory Comput..

[37] Ojeda-May P, Garcia ME (2010). Electric field-driven disruption of a native β-sheet protein conformation and generation of a helix-structure. Biophys. J..

[38] Baumketner, A. Electric field as a disaggregating agent for amyloid fibrils. *The J. Phys. Chem. B*. **118**, 14578–14589 (2014). 10.1021/jp509213f 25485693

[39] Worcester D. L. (1978). Structural origins of diamagnetic anisotropy in proteins.. Proceedings of the National Academy of Sciences.

[40] Gazit, E. A possible role for π-stacking in the self-assembly of amyloid fibrils. *The FASEB J*. **16**, 77–83 (2002). 10.1096/fj.01-0442hyp 11772939

[41] Inouye, H., Fraser, P. E. & Kirschner, D. A. Structure of beta-crystallite assemblies formed by Alzheimer beta-amyloid protein analogues: analysis by x-ray diffraction. *Biophys. J*. **64**, 502 (1993). 10.1016/S0006-3495(93)81393-6 PMC1262353 8457674

[42] Malinchik, S. B., Inouye, H., Szumowski, K. E. & Kirschner, D. A. Structural analysis of Alzheimer’s β (1–40) amyloid: protofilament assembly of tubular fibrils. *Biophys. J*. **74**, 537–545 (1998). 10.1016/S0006-3495(98)77812-9 PMC1299406 9449354

[43] Serpell, L. C., Blake, C. C. & Fraser, P. E. Molecular structure of a fibrillar Alzheimer’s Aβ fragment. *Biochemistry* **39**, 13269–13275 (2000). 10.1021/bi000637v 11052680

[44] Sikorski, P., Atkins, E. D. & Serpell, L. C. Structure and texture of fibrous crystals formed by Alzheimer’s Aβ (11–25) peptide fragment. *Structure* **11**, 915–926 (2003). 10.1016/s0969-2126(03)00149-7 12906823

[45] Lekprasert, B., Sedman, V., Roberts, C. J., Tedler, S. J. & Notingher, I. Nondestructive Raman and atomic force microscopy measurement of molecular structure for individual diphenylalanine nanotubes. *Opt. Lett*. **35**, 4193–4195 (2010). 10.1364/OL.35.004193 21165134

[46] Lambert, J., Shurvell, H., Lightner, D. & Cooks, R. Organic Structural Spectroscopy (Prentice-Hall. Inc., 1998).

[47] Le Bail Armel (2005). Whole powder pattern decomposition methods and applications: A retrospection. Powder Diffraction.

[48] Rodríguez-Carvajal, J. Introduction to the Program FULLPROF: Refinement of Crystal and Magnetic Structures from Powder and Single Crystal Data.

[49] Sánchez-Bajo, F. & Cumbrera, F. The use of the pseudo-Voigt function in the variance method of X-ray line-broadening analysis. *J Appl Cryst* **30**, 427–430 (1997).

[50] Liu, Y., Lin, J., Huang, G., Guo, Y. & Duan, C. Simple empirical analytical approximation to the Voigt profile. *JOSA B* **18**, 666–672 (2001).

[51] Zak, A. K., Majid, W. A., Abrishami, M. E. & Yousefi, R. X-ray analysis of ZnO nanoparticles by Williamson–Hall and size–strain plot methods. *Solid State Sci* **13**, 251–256 (2011).

[52] Ramakanth, K. *Basics of X-ray Diffraction and its Application* (I.K. International Pub. House, 2007).

[53] Kunitani, M. *et al*. Classical light scattering quantitation of protein aggregates: off-line spectroscopy versus HPLC detection. *J. Pharm. Biomed. Anal*. **16**, 573–586 (1997). 10.1016/s0731-7085(97)00191-x 9502153

