# Supplementary material for: Modulation of Peptide Based Nano-Assemblies with Electric and Magnetic Fields
Source: Sci Rep. 2017 Jun 2;7:2726. doi: 10.1038/s41598-017-02609-z (PMC5457452; doi:10.1038/s41598-017-02609-z)
Supplement: Supplementary file 1 — Supporting information [file 41598_2017_2609_MOESM1_ESM.pdf]

Supporting information

Modulation of Peptide Based Nano-Assemblies with Electric and Magnetic Fields

Gaurav Pandey, [†] *Jahnu Saikia,* [†] *Sajitha Sasidharan,* [†] *Deep C. Joshi,* [‡] *Subhash Thota,* [‡] *Harshal B. Nemade,* [§] *Nitin Chaudhary,* [†] *and Vibin Ramakrishnan* [†*]

[†]Department of Biosciences and Bioengineering, Indian Institute of Technology Guwahati, Guwahati-781039, India.

[‡]Department of Physics, Indian Institute of Technology Guwahati, Guwahati-781039, India.

[§]Department of Electronics and Electrical Engineering, Indian Institute of Technology Guwahati, Guwahati-781039, India.

[*]Email: vibin@iitg.ernet.in

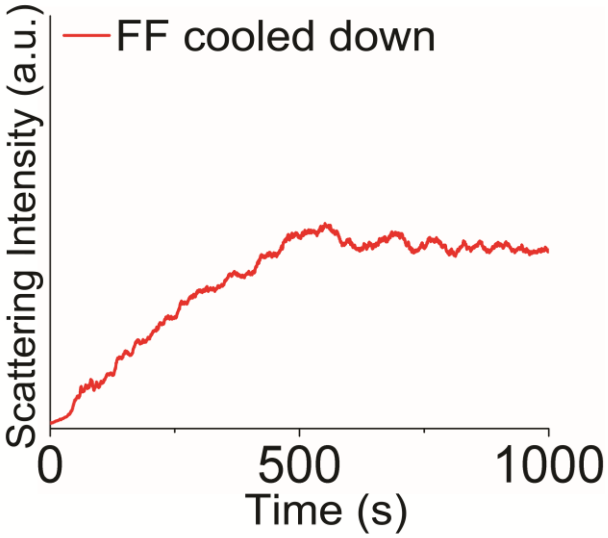

Figure S1 FF nano-assembly formation. Dissolved peptides were allowed to cool down from 95 ^{o}C to room temperature and concurrently static right angle scatter was recorded. The graph represents static right angle scatter plot in arbitrary units (a.u.).

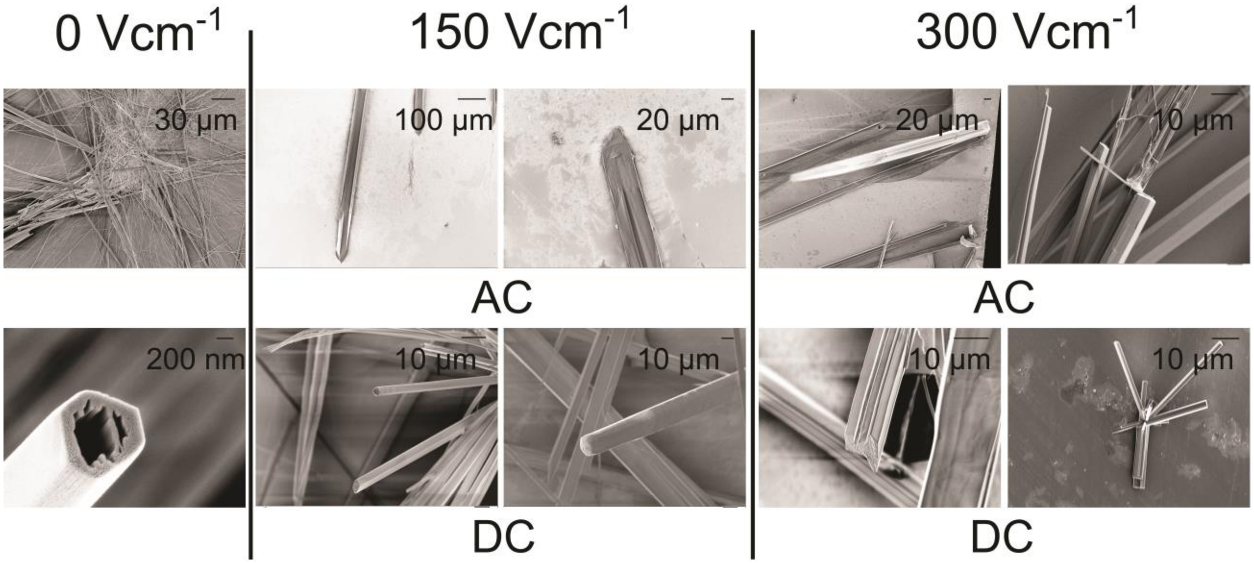

Figure S2 Scanning electron micrographs of FF self-assemblies formed post annealing at room temperature conditions: The nano rods and nanotubes formed after five experimental conditions reported (0 Vcm^{-1}, 150 Vcm^{-1} DC and AC electric field; 300 Vcm^{-1} AC and DC electric field) are shown. Field induced assemblies have greater tendencies of alignment and morphological shift to rod like structures (compared to tubes) in the overall population of nano-micro dimensional structures.

Table S1: Raman peak assignments for diphenylalanine self-assembled nanostructures.

Functional Group	Raman Spectra Peak (cm^{-1})
C-C<br>acyclic, aliphatic<br>stretching	1154,1300
Benzene ring	768,818,1004,1034,1590,1429,1608
C=O stretching	1688
CH stretching (aromatic)	3054
